# Influenza and Other Respiratory Viruses Involved in Severe Acute Respiratory Disease in Northern Italy during the Pandemic and Postpandemic Period (2009–2011)

**DOI:** 10.1155/2014/241298

**Published:** 2014-06-12

**Authors:** Elena Pariani, Marianna Martinelli, Marta Canuti, Seyed Mohammad Jazaeri Farsani, Bas B. Oude Munnink, Martin Deijs, Elisabetta Tanzi, Alessandro Zanetti, Lia van der Hoek, Antonella Amendola

**Affiliations:** ^1^Department of Biomedical Sciences for Health, University of Milan, Via C. Pascal 36, 20133 Milan, Italy; ^2^CIRI-IT, Department of Health Sciences, University of Genoa, Via A. Pastore 1, 16132 Genoa, Italy; ^3^Laboratory of Experimental Virology, Department of Medical Microbiology, Center for Infection and Immunity Amsterdam (CINIMA), Academic Medical Center of the University of Amsterdam, Meibergdreef 15, 1105 AZ Amsterdam, The Netherlands; ^4^Tehran University of Medical Sciences, 16 Azar Avenue, Enghelab Square, Tehran 1417614411, Iran

## Abstract

Since 2009 pandemic, international health authorities recommended monitoring severe and complicated cases of respiratory disease, that is, severe acute respiratory infection (SARI) and acute respiratory distress syndrome (ARDS). We evaluated the proportion of SARI/ARDS cases and deaths due to influenza A(H1N1)pdm09 infection and the impact of other respiratory viruses during pandemic and postpandemic period (2009–2011) in northern Italy; additionally we searched for unknown viruses in those cases for which diagnosis remained negative. 206 respiratory samples were collected from SARI/ARDS cases and analyzed by real-time RT-PCR/PCR to investigate influenza viruses and other common respiratory pathogens; also, a virus discovery technique (VIDISCA-454) was applied on those samples tested negative to all pathogens. Influenza A(H1N1)pdm09 virus was detected in 58.3% of specimens, with a case fatality rate of 11.3%. The impact of other respiratory viruses was 19.4%, and the most commonly detected viruses were human rhinovirus/enterovirus and influenza A(H3N2). VIDISCA-454 enabled the identification of one previously undiagnosed measles infection. Nearly 22% of SARI/ARDS cases did not obtain a definite diagnosis. In clinical practice, great efforts should be dedicated to improving the diagnosis of severe respiratory disease; the introduction of innovative molecular technologies, as VIDISCA-454, will certainly help in reducing such “diagnostic gap.”

## 1. Introduction

Most cases of influenza A(H1N1)pdm09 infection have a mild outcome; however some present as severe acute respiratory infection (SARI) and require admission to intensive care unit (ICU) [1, 2]. The main reason for admission to ICU is a pulmonary inflammatory syndrome characterized by diffuse alveolar damage (acute respiratory distress syndrome: ARDS), which can be fatal. Since the beginning of 2009 pandemic, international health authorities recommended monitoring severe and complicated cases of influenza infection [3, 4]. Considering the serious outcome of these respiratory diseases, the contribution of other respiratory pathogens besides A(H1N1)pdm09 should be envisaged [5]. Additionally, in clinical practice, a specific causative agent which explains the respiratory symptoms is often unidentified, owing to the lack of sensitive tests or the presence of an as-yet unknown pathogen. The recently developed VIDISCA-454 (*VIrus DIScovery using CDNA Amplified fragment-length polymorphism* combined with* Roche-454* high-throughput sequencing) is a sensitive sequence-independent virus discovery technique which can be used to reveal as-yet unknown viruses [5, 6].

This study aimed at evaluating the proportion of SARI/ARDS cases and deaths due to A(H1N1)pdm09 infection and assessing the impact of other respiratory pathogens during pandemic and postpandemic period (2009–2011) in northern Italy as well as searching for unknown viruses in those cases for which diagnosis remained negative. To this end, common respiratory pathogens were investigated and VIDISCA-454 methodology was applied on samples which remained negative for all tested pathogens.

## 2. Materials and Methods

In the capacity of reference laboratory operating within InfluNet network [7], our laboratory is in charge of carrying out the virological surveillance of severe forms of influenza infection in Lombardy (nearly 10 million inhabitants). From October 1, 2009, to April 30, 2011, 206 respiratory samples were collected from patients hospitalized due to severe respiratory illness. Of these, 61.2% were males with a median age of 44.3 years (IQR: 49.7 years; range: 1 month–89 years); 17.5% were children ≤ 5 years and 23.3% were ≥65 years. Data on comorbidities presence were available for nearly 70% of study patients: 64.3% reported medical conditions [3, 4]; in detail, 25.6% had weakened immune system (due to cancer, HIV/AIDS, or long-term steroid treatment), 19.7% heart disease, 11.6% asthma/chronic lung disease, and 10.4% neurological/neurodevelopmental conditions. Out of 206 patients, 91 (59.3% males; 18.7% aged ≤ 5 years, 58.2% aged 6–64 years) were SARI cases who required admission to ICU and extracorporeal membrane oxygenation (ECMO) therapy, and 115 (62.6% males; 16.5% aged ≤5 years, 60% aged 6–64 years) were ARDS cases, as defined by the European Consensus Conference [8]. Nine ARDS patients (median age: 35.6 years, IQR: 21.4 years) died during hospitalization: case fatality rate (CFR) in our ARDS series was 7.8% (9/115). No SARI case was fatal.

Respiratory specimens (paired nasal/oral swab and bronchoalveolar lavage) were collected from each SARI/ARDS case. Nucleic acids were purified by* NucliSENS easyMAG* (*bioMérieux,* France) and analyzed by real-time RT-PCR assay to identify influenza virus. In detail, a one-step real-time RT-PCR assay was performed to simultaneously detect influenza viruses type A and B [9]. The subtyping of influenza A positive samples was carried out by a one-step real-time RT-PCR assay using specific primer/probe sets for the hemagglutinin gene [10].

The clinical specimens that resulted negative to influenza virus detection were then screened by real-time RT-PCR/PCR for a panel of respiratory pathogens (*Respiratory MWS r-gene Real-time PCR, bioMérieux*, France) to detect respiratory syncytial virus (RSV) A and B; human metapneumovirus (hMPV) A and B; human rhinovirus (hRV) and enterovirus (hEV); adenovirus (AdV); human bocavirus (hBoV) 1–4; human coronavirus (hCoV) 229E, NL63, OC43, HKU1; human parainfluenza virus (hPIV) 1–4;* Chlamydophila pneumoniae; Mycoplasma pneumoniae.*

Cases which resulted negative to all diagnostic assays were further investigated by VIDISCA-454 technique. This is a virus discovery method based on recognition of restriction enzyme cleavage sites, ligation of adaptors, and subsequent amplification by PCR combined with high-throughput sequencing 454 FLX/Titanium system (Roche, USA) [5].

## 3. Results

Influenza A(H1N1)pdm09 virus was detected in 58.3% (120/206) of SARI/ARDS cases (61.7% males; 13.3% aged ≤ 5 years, 67.5% aged 6–64 years). Moreover, the presence of another condition possibly increasing the risk for developing influenza-related complications [3, 4] was acknowledged for nearly half of A(H1N1)pdm09-positive SARI/ARDS cases: 25.4% had weakened immune system, 15.2% had heart disease, 11.9% were morbidly obese people (body mass index ≥ 40), 10.2% had asthma/chronic lung disease or neurological/neurodevelopmental conditions, and 4.2% were pregnant women. Approximately half (62/120: 51.7%) of A(H1N1)pdm09-positive patients had ARDS. It is worth mentioning that A(H1N1)pdm09 was identified in 77.8% (7/9) of fatal ARDS cases (42.9% males, median age: 30.4 years, IQR: 15.4 years). Four (4/7: 57.1%) of these individuals belonged to risk categories (i.e., two were cancer patients, one was morbidly obese, and one had underlying neurodevelopmental conditions). Thus, the A(H1N1)pdm09 CFR was 11.3% (7/62).

The impact of respiratory pathogens other than A(H1N1)pdm09 was 19.4% (40/206) (65% males; 30% aged ≤ 5 years, 47.5% aged 6–64 years). HRV/hEV were the most frequently identified viruses followed by influenza A(H3N2) virus, accounting for 27.5% (11/40) and 20% (8/40) of infections, respectively (Table 1). No fatal cases were ascribable to pathogens other than A(H1N1)pdm09.

**Figure 1:**
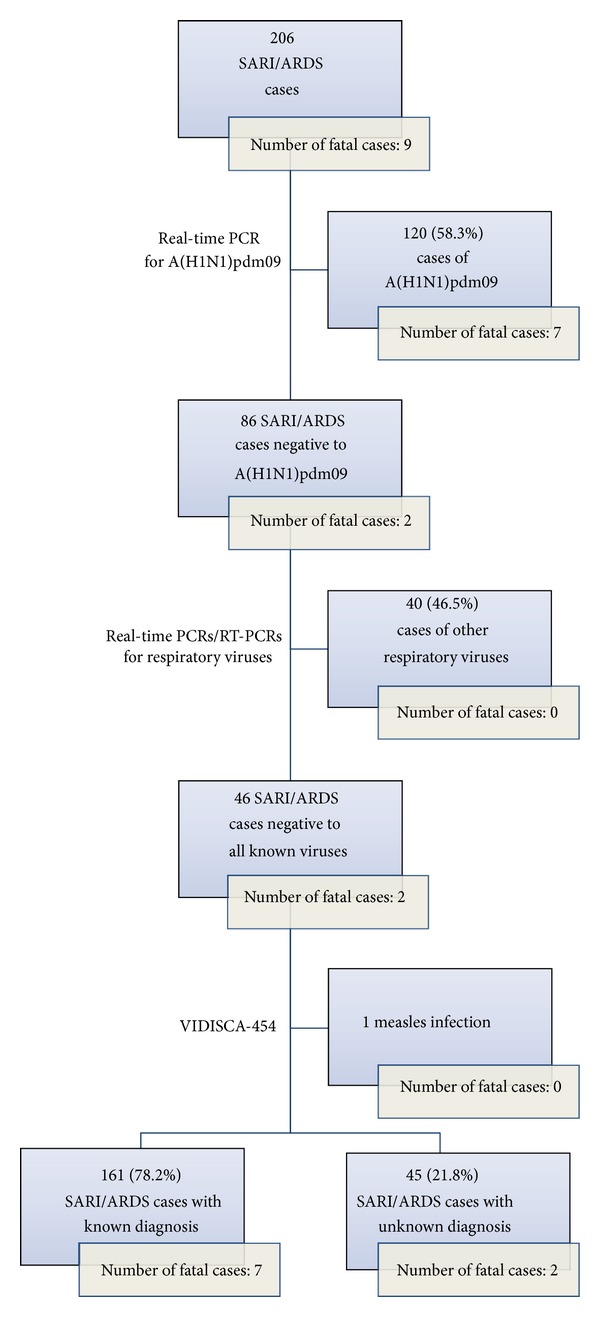
Study design and results overview.

Forty-six (46/206: 22.3%) SARI/ARDS cases (including two fatalities) resulted negative to all diagnostic assays (58.2% males; 18.2% aged ≤ 5 years, 45.4% aged 6–64 years) and were further investigated by VIDISCA-454 [5, 11]. VIDISCA-454 revealed no sequence reads that could belong to a novel virus or viral variant in any of the 46 specimens; however it enabled the identification of one case of undiagnosed measles, thus increasing the proportion of cases with a diagnosis to 78.2% (161/206). Hence, the overall proportion of cases with unknown diagnosis was 21.8% (45/206); most (34/45: 75.6%) cases that could not be diagnosed were ARDS and two (2/45: 4.4%) were fatal.  Figure 1 summarizes study results.

## 4. Discussion

During pandemic and postpandemic period, several pathogens cocirculated and were associated to severe respiratory infections; however, influenza A(H1N1)pdm09 virus had the greatest impact (58.3%) in our SARI/ARDS series. More than half (51.7%) of A(H1N1)pdm09 infection resulted in ARDS. It is interesting to note that most (67.5%) severe respiratory diseases due to A(H1N1)pdm09 infection were identified among 6–64-year-old individuals. The A(H1N1)pdm09 case fatality rate in our ARDS series was 11.3% fatal cases in young adults, and 42.8% did not belong to any at-risk category [3, 4]. This data is in agreement with other studies; Van Kerkhove et al. have reported a median age of 46 years among fatal laboratory-confirmed A(H1N1)pdm09 cases [12]. McCallum has described that during the 2009 pandemic only 1% of laboratory-confirmed cases and 13% of laboratory-confirmed deaths were among persons 65 years of age or older [13]. The Global Pandemic Mortality (GLaMOR) project has evaluated that although the pandemic mortality estimate was similar in magnitude to that of seasonal influenza, a marked shift toward mortality among persons 65 years of age occurred, so that many more life-years were lost [14]. Such an age shift has been documented as well by several studies on A(H1N1)pdm09 mortality [15–17].

The proportion of SARI/ARDS cases associated with respiratory viruses other than A(H1N1)pdm09 was significantly lower (19.4% versus 58.3%, *P* value <0.0000001). Severe respiratory diseases associated with respiratory viruses other than A(H1N1)pdm09 were detected more frequently among children ≤ 5 years (13.3% versus 30%, *P* value = 0.02). This piece of evidence is in accordance with the results of other studies reporting a notable burden of respiratory viruses in children under 5 years [18–20].

Studies published to date have suggested that influenza viruses and rhinoviruses are the leading causes of severe respiratory disease leading to hospitalization [21, 22], similarly to what was observed in our SARI/ARDS series, where hRV/hEV were the most common identified viruses along with influenza viruses. Also influenza A(H3N2) virus played a significant role in our SARI cases and caused ARDS in one patient with a weakened immune system due to HIV/AIDS. Overall, the proportion of SARI/ARDS correlated to an influenza A virus infection was 62.1% (128/206), thus emphasizing the central role of influenza A virus in severe respiratory infection [23, 24].

The use of molecular assay has notably contributed to identifying pathogens possibly involved in severe respiratory disease, thus allowing getting to a diagnosis of viral infection in nearly 80% of study SARI/ARDS cases. Other studies that have not used nucleic acid amplification assays have typically reported that 5–20% of cases of acute respiratory infection have a viral etiology [25]. In addition, it is noteworthy that VIDISCA-454 enabled the identification of one measles infection that escaped clinical diagnosis in one SARI case. Hence, measles infection should be considered in complicated pulmonary disease, as also suggested by others [26], since measles virus is not generally included in respiratory diagnostic panels.

## 5. Conclusions

During pandemic and postpandemic period, several pathogens cocirculated and were associated to severe respiratory infections, with influenza A(H1N1)pdm09 virus having the greatest impact. Nearly 22% of SARI/ARDS cases did not obtain a definite diagnosis, and among these cases two were fatal. In clinical practice, great efforts should be devoted to improving diagnosis of severe respiratory infections and to reducing such “diagnostic gap.” The advantage from relying upon more accurate diagnosis could benefit the patient, in terms of receiving the more appropriate antiviral drugs, and could provide more detailed information on viruses circulating in the community, thus making public health authorities aware so as to adjust their policies accordingly.

VIDISCA-454 proved to be a sensitive and specific methodology that can be successfully applied to surveillance of viral respiratory infections that represent an ever-changing field due to the continuous emergence of new viruses (i.e., influenza A(H5N1) and A(H7N9) viruses, MERS-CoV).

## Figures and Tables

**Table 1:** Impact of respiratory pathogens on the patients with SARI/ARDS (*N* = 206).

Pathogen	Number of positive samples		
	SARI (*N* = 91)	ARDS (*N* = 115)	SARI/ARDS (*N* = 206)
Influenza A(H1N1)pdm09 virus	58	62	120
hRV/hEV	7	4	11
Influenza A(H3N2) virus	7	1	8
RSV	3	1	4
hCoV	2	2	4
hPIV	2	2	4
AdV	2	0	2
Influenza B virus	1	0	1
hBoV	1	0	1
hMPV	0	0	0
*Chlamydophila pneumoniae *	0	0	0
*Mycoplasma pneumoniae *	0	0	0

Coinfections	2	3	5

## References

[1] Larussa P (2011). Pandemic novel 2009 H1N1 influenza: what have we learned?. *Seminars in Respiratory and Critical Care Medicine*.

[2] Swerdlow DL, Finelli L, Bridges CB (2011). 2009 H1N1 influenza pandemic: field and epidemiologic investigations in the united states at the start of the first pandemic of the 21st century. *Clinical Infectious Diseases*.

[3] European Center for Disease Control (ECDC) Surveillance of severe disease due to influenza in Europe. http://www.ecdc.europa.eu/en/healthtopics/seasonal_influenza/Documents/1201_ECDC_concept_paper_Surveillance_of_severe_disease_due_to_influenza_in_Europe.pdf.

[4] Italian Ministry of Health Surveillance of severe forms of influenza A(H1N1)pdm09 infection. http://www.trovanorme.salute.gov.it/normsan-pdf/0000/31217_1.pdf.

[5] de Vries M, Deijs M, Canuti M (2011). A sensitive assay for virus discovery in respiratory clinical samples. *PLoS ONE*.

[6] van der Hoek L, Pyrc K, Jebbink MF (2004). Identification of a new human coronavirus. *Nature Medicine*.

[7] InfluNet http://www.iss.it/iflu/.

[8] Bernard GR, Artigas A, Brigham KL (1994). The American-European Consensus Conference on ARDS: definitions, mechanisms, relevant outcomes, and clinical trial coordination. *The American Journal of Respiratory and Critical Care Medicine I*.

[9] World Health Organization (WHO) Global Influenza Surveillance Network (GISN) Manual for the laboratory diagnosis and virological surveillance of influenza. http://whqlibdoc.who.int/publications/2011/9789241548090_eng.pdf.

[10] Center for Disease Control (CDC) 2009 protocol CDC protocol of real-time RT PCR for swine influenza A(H1N1). http://www.who.int/csr/resources/publications/swineflu/CDCrealtimeRTPCRprotocol_20090428.pdf.

[11] van der Hoek L, de Vries M, Munnink BBO (2012). Performance of VIDISCA-454 in feces-suspensions and serum. *Viruses*.

[12] van Kerkhove MD, Vandemaele KAH, Shinde V (2011). Risk factors for severe outcomes following 2009 influenza a (H1N1) infection: a global pooled analysis. *PLoS Medicine*.

[13] McCallum L (2010). Epidemiological characteristics of the influenza A(H1N1)2009 pandemic in the Western Pacific Region. *Western Pacific Surveillance and Response*.

[14] Simonsen L, Spreeuwenberg P, Lustig R, Taylor RJ, Fleming DM, Kroneman M (2013). Global mortality estimates for the 2009 Influenza Pandemic from the GLaMOR project: a modeling study. *PLoS Medicine*.

[15] Charu V, Chowell G, Mejia LSP (2011). Mortality burden of the A/H1N1 pandemic in Mexico: a comparison of deaths and years of life lost to seasonal influenza. *Clinical Infectious Diseases*.

[16] Lemaitre M, Carrat F, Rey G, Miller M, Simonsen L, Viboud C (2012). Mortality burden of the 2009 A/H1N1 influenza pandemic in France: comparison to seasonal influenza and the A/H3N2 pandemic. *PLoS ONE*.

[17] Muscatello DJ, Cretikos MA, MacIntyre CR (2010). All-cause mortality during first wave of pandemic (H1N1) 2009, New South Wales, Australia, 2009. *Emerging Infectious Diseases*.

[18] Rudan I, 'Brien KL O, Nair H, Liu L, Theodoratou E, Qazi S (2013). Epidemiology and etiology of childhood pneumonia in 2010: estimates of incidence, severe morbidity, mortality, underlying risk factors and causative pathogens for 192 countries. *Journal of Global Health*.

[19] Walker CLF, Rudan I, Liu L (2013). Global burden of childhood pneumonia and diarrhoea. *The Lancet*.

[20] Verani JR, McCracken J, Arvelo W, Estevez A, Lopez MR, Reyes L (2013). Surveillance for hospitalized acute respiratory infection in Guatemala. *PLoS One*.

[21] Jennings LC, Anderson TP, Beynon KA (2008). Incidence and characteristics of viral community-acquired pneumonia in adults. *Thorax*.

[22] Marcos MA, Camps M, Pumarola T (2006). The role of viruses in the aetiology of community-acquired pneumonia in adults. *Antiviral Therapy*.

[23] Wiemken T, Peyrani P, Bryant K (2013). Incidence of respiratory viruses in patients with community-acquired pneumonia admitted to the intensive care unit: results from the Severe Influenza Pneumonia Surveillance (SIPS) project. *European Journal of Clinical Microbiology and Infectious Diseases*.

[24] Choi SH, Hong SB, Ko GB, Lee Y, Park HJ, Park SY (2012). Viral infection in patients with severe pneumonia requiring intensive care unit admission. *The American Journal of Respiratory and Critical Care Medicine*.

[25] File TM (2003). Community-acquired pneumonia. *The Lancet*.

[26] Do AHL, van Doorn HR, Nghiem MN (2011). Viral etiologies of acute respiratory infections among hospitalized vietnamese children in Ho Chi Minh City, 2004–2008. *PLoS ONE*.

